# Mind Cure, No. 3

**Published:** 1888-07

**Authors:** 


### MIND CURE NO. 3.

### CONCLUDED.

### VI.

The soul partakes of the essence of the nature of Deity. The soul has no determined place in the body, but the whole of it is in every part. Matter is divisible; spirit indivisible; matter is changeable and corrupted. Mind is a pure substance. Mind is active or possesses force; matter is passive, inert, the thing acted upon. The mind has a personal identity; particles of the body are continually changing.

Every emotion of the mind has its peculiar nerve upon which it acts. Through emotion secretions not only become more copious, but are essentially changed in quality. The saliva and milk become poisons. Habits are conscious and unconscious. Habits are periodical; some returning at months, and even years.

The power of the will is stronger than the strongest habit. Fear relaxes the muscles and strips the whole system of energy. A strong will and courage have the opposite effect; the mind affects every organ of the body. Vice and crimes are known to produce deformity of the heart. Testa found the heart of a hardened criminal was hard, hairy and skinny. Riold found the heart of a vicious man gristly.

Chrildren have no motives; you are not responsible for your children ; they are all held by a strong hand ; you are not responsible for their lives.

VII.

The truth carries us—not we the infinite truth ; in confidence shall be my strength ; we can never lack confidence, for our sufficiency is from God. Remember your strength is to sit still, and in all doubtful emergencies wait silently until self is silenced and the mind is empty of material thought, and then let God pour his thoughts into your mind and write his law upon your heart. Empty yourself of sympathies so as to have no weak spot where the evil thoughts (diseases) of others can enter, as they are unreal. The thought is the real thing ; be full of divine compassion. If I have a weak spot, or the old sympathy, my patient makes an image for my mind, and I respond to her false thought, not she to my perfect thought.

I can never take on a belief of disease, for there is nothing in me to perceive it, and surely nothing in me to mirror it, for I am an expression or manifestation of infinite life and infinite substance.

VIII.

Cast yourself upon the other mind, casting your false thoughts away and open your thoughts to the infinite mind. It is unsafe to say you are tired ; in admitting this falsity you open the door for all the other false images. Every belief, fear of cold, brings its fear of consumption, a law man made generations back. The mind expresses itself every moment Whatever thoughts you hold must be expressed. If you have a man's ruling idea, his central love, the desire of his life, you have the whole man ; all his thoughts and feelings revolve around these. Matter has the rest of its being in Infinite mind, and without that it has no existence. Matter to spirit is invisible. The body interprets the mind. A man's physical characteristics are the fixed modes of thought ; the feelings and sensations of the body are in the mind ; the strength or weakness of the body is in the mind ; its health or disease depends upon the mind. Matter is not evil ; there is no boundary line between matter and spirit ; an idea is the thing itself. The idea man, held in infinite mind, is man in reality ; strength is not a bodily condition, but a mental force.

IX.

Soul is the rational, spiritual and immortal part in man ; the seat of feeling in distinction from intellect. It is also a quality of mind. Metaphysics is that which regards the ultimate grounds of being, as distinguished from the phenomenal. The fundamental law of mathematics and metaphysics is identity; the law of identity is the same yesterday, to-day and forever—the law by which one equals one and never two.

Directions for Self-treatment :—The impression I have is a lie, and I will not believe it. What is there of me to have pain? Nothing but life and forms of life, and life holds no suffering; this false idea of pain cannot remain in my mind if I do not nourish it by believing in it. An expression of disease cannot grow without nourishment. I nourish it by believing it; when I deny it and repudiate it and cast it aside, it has no power over me. I am a manifestation of harmony, and cannot contain discord. I am perfectly well, and always have been. I deny all power to the thought of pain, or disease held in the thoughts about me. I desire and claim only an inflow of thought from the Infinite intelligence, and from that source comes only ideas of life and peace, joy, harmony and rest. I am a perfectly well, happy and pure spirit, and will hold these thoughts of myself not in my own right, but by simply resting in the faith that my substance and life are perfection.

Directions for Treating :—Ascertain, firstly, what has produced the appearance of sickness in your patient. There is no cause of sickness outside of fear. Ask what fright they have ever had in their lives; grief is only a form of fear. If they cannot recall any fright, ask them if they have had some deep grief; belief in inheritance is a very common fear; sometimes you can trace the morbid idea by asking what kind of reading they enjoy the most; find out in some way what produces the greatest fear. Be deliberate and careful in all questioning; say always to yourself, this individuality is not sick; he is holding on to the morbid belief which is to be rooted out of his mind. Make yourself calm and receptive to the spirit of truth.

Let them tell you at once what is the cause of their apparent illness; all causes, to them, will be natural causes, no matter what they tell you; remember that back of what they say is the mental cause or the erroneous judgment. Never for a moment, realize the material plane of being; regard all the causes of what they will tell you as simple dreams, and make them such in your silent argument. When you have made the circumstances which they think the cause of their illness a dream, the effect or illness will leave the body. Strive to have your thoughts as concentrated as possible, that the patient may receive a clear mental picture. You are not to think of them as within the body; they are both within and outside the body.

Forms of Treatment :—First, be quiet ; then say mentally—be still, and know that I am God ; I am the temple of the living God ; the Lord is in his holy temple ; let all the earth keep silent before him. Centre yourself in God. Say God made all there is, and nothing was made that he did not make ; he called it good and saw that it was good ; therefore, it is perfect and harmonious. I am one with God ; nothing can separate me from God ; he manifests himself through me ; my strength cannot fail ; my sufficiency is in God.

*Second Step :*—When you are still and can realize that unto God nothing is impossible, think of your patient ; realize that he is within you and holds the same relation to God that you do. In the treatment soul meets soul, and for the time the patient lives through the inspired thoughts held in your mind. Do not think of the perfect spiritual body, but of the perfect idea held in the mind of God. Think your thoughts in a quiet, orderly manner, and describe to him his own identity (that is, his perfect state of health) as an artist would describe a picture held in his mind ; prove to him that he is independent of his body ; prove to him that he can only inherit from one source. The patient's own existence is only in the sphere of causes—Infinite mind is a grand whole ; organs are only symbols of thought and affection, and are expressed in form only in the world of effects ; the spiritual world is also a world of effects ; therefore, he is receptive only to Infinite intelligence, and that could never report discord or falsity ; therefore, the patient is ignorant of the falsities his phenomenon expresses. Say to your patient—you know you are well, and always have been, and nothing can make you think differently.

*Third Step :*—Then again turn your mind (still holding the patient's identity) to God, and say : I leave you a naked soul in the heart of God. I know you are perfectly safe and well ; that even this phenomenon expresses a perfect thought of God and every function must be orderly because it is only subject to God's laws.

*Fourth Step* :—Nothing can obstruct God's law. I ask nothing ; I simply receive the knowledge that God gave me ; that he is omnipotent and shows it forth through me and through this other soul ; I rest in the Lord, and know that he will give me the desire of my heart.

Thoughts from Mrs. Stuart :—The senses will tell us there is an intense beating in the head ; also suggest a fever and nausea from the external plane of our being ; we will begin to look for some external cause, *i.e.* indigestion, etc. You must turn your mind at once, and ask yourself this question: What is there in my head to ache? There is nothing there but life which comes from the great source of all reality, and this life is not subject to pain or inharmony of any kind. We can believe a falsehood or we can believe a truth. The external senses, always tell you a falsehood. Listen to your spiritual thoughts and say to yourself that life cannot include sickness, and that all that pertains to life is eternal and unchangeable; say to the presence of pain or to your thoughts: you do not belong to me, and I know that you cannot exist without my recognition.

I recognize only that I am spiritual, and that I am fed, nourished, and controled by spirit. Treatment is simply showing the truth, and when you realize that truth works for itself, you will see that anxiety retards the work.

FORMS OF SELF-TREATMENT:—Earth means in the old thought the carnal mind. In mind cure, Heaven means the interior and spiritual or celestial state. When anything that annoys or troubles, pains or frightens, distresses mentally or physically, say I am spirit not matter; this body is the instrument of man on the material plane; my instrument, the expression of my will and thought, but not my real self, myself. My identity exists in all perfection, in the Mind of the Infinite; as an idea of that mind it partakes of the love, truth and power of that mind. All love and expressions of love exist in me, and I in them. As I am an expression of truth I can only express truth. As I am an expression of Infinite power, so all power in Heaven and Earth belongs to me as spirit. I can never lack confidence in this power, for my sufficiency is of God; whatever appears to me of discord, or in harmony in myself or others, is an illusion; it is an expression of erroneous idea, and will disappear when the false idea is dispersed by truth. I claim for this body, phenomenon, an external expression of myself, all harmony, and that is all I do claim; power, life, thought belongs to spirit. Power, thought and life belong to spirit, and I now and hereafter so order my thoughts, that the light, life and truth within me, the Lord himself—i. e. all good itself—can flow through to the ultimate, even my body, without check or hindrance. I live in the Infinite life, enjoy in the Infinite love, and think only in Infinite truth; I deny all else. I am receptive only to the Infinite, so no thoughts of evil or falsity shall touch me as I rest securely in Divine life. All outside is darkness, and I cannot perceive anything beyond the light.

I deny to the false thoughts within me all will or intelligence. I have no mortal parents for in mortality there is no life. I am immortal, and inherit only from the source of immortal life. I wake from my old thought of self as I would from a dream. My life in that sense has always been a dream. I know now where I am and what I am. I rest in confidence ; I will be silent and listen. I withdraw myself from the false life of the external and find my centre ; from the interior comes my knowledge ; I accept no other teachings ; I am spirit, and will live now and think only as spirit. I cannot be deceived. Every idea of suffering, pain, or evil, comes from the false, perishable, mental thought ; every form of self-love comes from the same source. I give myself utterly unto Infinite conciousness, and claim nothing as from myself. I utterly lose myself in an abyss of life, love and power, and will know nothing beyond ; I in God, he in me, and with his Divine life, intelligence and love, always striving for outward expression in me as a part of the immortal soul ; I know that all there is of reality in me is perfection, and I know that I have all the old falsities in thought. Every organ in this phenomenal body will express perfection. I am well ; and as in spirit there is no time, what is now is eternal. I am the temple of the living God ; the Lord is in his temple ; let all the earth keep silence before him.

